# Visual Magnetic Resonance Imaging (MRI) Grading of Thigh Muscle Fatty Infiltration and Bulk Loss in Elderly Patients With Chronic Knee Pain

**DOI:** 10.7759/cureus.97981

**Published:** 2025-11-27

**Authors:** Muhannad N Alharbi, Abdullah A Altowairqi

**Affiliations:** 1 Radiology, Prince Sultan Military Medical City, Riyadh, SAU

**Keywords:** chronic knee pain, elderly patients, fatty infiltration, muscle atrophy, muscle bulk loss, muscle degeneration, periarticular muscles

## Abstract

Background: Chronic knee pain is highly prevalent among older adults and contributes substantially to mobility limitation and reduced quality of life. Emerging evidence suggests that periarticular muscle degeneration, particularly fatty infiltration and atrophy, plays an important role in symptom severity and functional decline. Magnetic resonance imaging (MRI) allows direct visualization of muscle quality, and semi-quantitative visual grading may offer a practical approach suitable for routine clinical practice.

Objective: To evaluate thigh and peri-knee muscle quality using a semi-quantitative MRI visual grading system in elderly patients with chronic knee pain and to examine its association with pain severity and functional limitation.

Methods: This cross-sectional study included 150 elderly patients who underwent routine MRI of the knee and the corresponding thigh. Fatty infiltration and muscle bulk were graded visually on a 0-3 scale for major muscle groups, including the quadriceps and hamstrings. Subcutaneous fat prominence was similarly assessed. Pain severity and functional limitation scores were recorded. Pearson correlations were used to assess associations between MRI scores and clinical measures.

Results: The mean age was 65.6 ± 6.3 years, and 54.7% were women. Quadriceps muscles demonstrated the greatest degree of fatty infiltration (mean score 1.7 ± 0.8), with 58% showing moderate to severe changes. Quadriceps bulk loss was observed in 55% of participants. Visual muscle scores showed significant correlations with pain severity and functional limitation, with the strongest associations observed for quadriceps fatty infiltration (r = 0.46-0.51) and quadriceps bulk loss (r = 0.49-0.53). Subcutaneous fat prominence demonstrated weaker but statistically significant correlations.

Conclusion: Semi-quantitative visual MRI assessment reveals substantial muscle degeneration among elderly patients with chronic knee pain, particularly involving the quadriceps. These visual findings correlate strongly with clinical severity and demonstrate high reproducibility, supporting their utility in routine MRI interpretation. Incorporating systematic muscle evaluation into standard knee MRI may help identify patients at risk for functional decline and inform targeted rehabilitation strategies.

## Introduction

Chronic knee pain is one of the most common musculoskeletal complaints among older adults and a major contributor to mobility limitation and reduced quality of life worldwide. Its prevalence increases with age and cardiometabolic comorbidities, placing a substantial burden on healthcare systems in aging populations [1,2]. In Saudi Arabia, community-based surveys indicate high rates of symptomatic knee problems among adults over 60 years of age, with increasing clinical demand across tertiary centers across the kingdom [3]. Despite the widespread use of radiography and conventional MRI, early identification of patients at risk for functional decline remains challenging, as imaging has traditionally focused on cartilage, bone, and meniscal pathology.

Growing evidence suggests that periarticular muscle degeneration plays a critical role in the development and progression of chronic knee symptoms. Factors such as pain-related disuse, neural inhibition, and age-associated sarcopenia contribute to muscle atrophy, fatty infiltration, and reduced strength, collectively described as arthrogenic muscle changes [4-6]. These alterations may worsen joint loading patterns, reduce stability, and impair gait, thereby intensifying symptom severity. Among the various muscular changes, intramuscular fatty infiltration has gained attention as a potential imaging marker linked to frailty, metabolic dysfunction, and diminished physical performance in older adults [6-8].

Magnetic resonance imaging (MRI) enables direct visualization of muscle composition and quality. While advanced quantitative techniques exist, prior studies have shown that semi-quantitative visual assessment can reliably characterize fatty infiltration and bulk loss in routine clinical settings [7,9]. These practical scoring methods are particularly useful in centers where specialized fat-water sequences are unavailable. Previous research, primarily from Western cohorts, has demonstrated associations between visually assessed muscle degeneration and pain severity or mobility impairment, but data from Middle Eastern populations remain limited, and few studies have evaluated multiple thigh and hip stabilizer muscle groups together [8-11].

A better understanding of MRI-detected muscle changes in patients with chronic knee pain may help refine disease characterization, identify individuals at higher risk of functional deterioration, and guide early rehabilitative strategies. Therefore, the present study aimed to evaluate thigh and peri-knee muscle quality using a semi-quantitative MRI visual grading system in elderly patients with chronic knee pain and to examine its association with pain severity and functional limitation.

## Materials and methods

Study design and participants

This cross-sectional study included 150 elderly patients presenting with chronic knee pain. Consecutive patients who underwent MRI of both the knee and thigh as part of their clinical evaluation were eligible. Exclusion criteria included previous knee or thigh surgery, acute trauma, infection, inflammatory arthropathy, or known neuromuscular disorders. Demographic and basic clinical information, including age, sex, BMI, diabetes status, symptom duration, and pain severity, was recorded at the time of imaging.

MRI acquisition

All participants underwent MRI of the affected knee and corresponding thigh using a 1.5-T system. Standard routine sequences used in our institution were acquired, including axial and sagittal T1-weighted images, T2-weighted images with fat suppression, and proton density (PD) fat-saturated sequences. Imaging coverage included the entire thigh to knee to allow evaluation of major muscle groups (quadriceps and hamstrings).

Semi-quantitative muscle assessment

Muscle quality was analyzed using a semi-quantitative visual grading system applied to all thigh MRI studies. Fatty infiltration for each muscle group was graded on a 4-point scale (0 = none, 1 = mild, 2 = moderate, 3 = severe) based on the proportion and distribution of intramuscular fat on T1-weighted images. Muscle bulk was similarly graded on a 4-point scale reflecting the degree of muscle size reduction relative to expected age-matched appearance. Subcutaneous fat prominence was assessed visually on a 0-3 scale. Visual assessment was performed on a normal subject to evaluate subcutaneous fat and to grade fatty infiltration according to the Goutallier classification.

Statistical analysis

Descriptive statistics were generated for demographic and imaging variables. Correlations between MRI visual scores and clinical parameters (pain and functional limitation) were examined using Pearson correlation coefficients. Group comparisons were analyzed using standard statistical tests, with significance set at p < 0.05. Analyses were performed using IBM Corp. Released 2017. IBM SPSS Statistics for Windows, Version 26.0. Armonk, NY: IBM Corp.

## Results

Study population

A total of 150 elderly participants were included. The mean age was 65.6 ± 6.3 years, and 54.7% were women. The mean BMI was 27.8 ± 4.3 kg/m², and 24% of the cohort had diabetes mellitus. Right and left knee involvement was nearly balanced (78 vs. 72 cases). Physical activity levels varied across the cohort, with 52 patients reporting low activity, 70 with moderate activity, and 28 with high activity. The median duration of symptoms was 6.2 years (IQR, 3.5-9.1). Baseline characteristics are shown in Table 1.

**Table 1:** Baseline characteristics of the study population (n = 150) This table summarizes demographic and clinical characteristics of the 150 elderly patients included in the study, including age, sex distribution, BMI, comorbidities, symptom duration, and pain severity. BMI: Body mass index, VAS: Visual Analogue Scale

Variable	Total (n = 150)
Age, years (mean ± SD)	65.6 ± 6.3
Female sex, n (%)	82 (54.7)
BMI, kg/m² (mean ± SD)	27.8 ± 4.3
Diabetes mellitus, n (%)	36 (24.0)
Side affected (Right/Left)	78 / 72
Physical activity (low/moderate/high)	52 / 70 / 28
Symptom duration, years (median, IQR)	6.2 (3.5–9.1)
Pain VAS 0–10 (mean ± SD)	6.3 ± 1.9

Semi-quantitative MRI muscle assessment

All patients underwent MRI of both the knee and thigh. Visual evaluation demonstrated a consistent pattern of muscle quality changes across major muscle groups. Quadriceps muscles showed the highest degree of fatty infiltration (mean score 1.7 ± 0.8). More than half of the patients (58%) exhibited moderate to severe quadriceps fatty infiltration. Muscle bulk loss followed a similar distribution, with quadriceps bulk reduction observed in 55% of patients. Subcutaneous fat prominence was also common, with 60% showing moderate to severe visual grading. Detailed MRI findings are presented in Table 2.

**Table 2:** Semi-quantitative MRI muscle assessment using visual grading This table presents mean visual scores for fatty infiltration, muscle bulk loss, and subcutaneous fat prominence across major muscle groups, along with the proportion of patients classified as having moderate to severe findings (score ≥ 2).

MRI Variable (Visual Assessment)	Mean Score ± SD	% Moderate–Severe (Score ≥ 2)
Quadriceps fatty infiltration	1.7 ± 0.8	58%
Quadriceps muscle bulk loss	1.6 ± 0.9	55%
Subcutaneous fat prominence (0–3 scale)	1.8 ± 0.9	60%

Correlation between MRI findings and clinical severity

There were significant correlations between visually assessed muscle quality and both pain severity and functional limitation. Higher fatty infiltration and greater loss of muscle bulk were associated with worse functional status. Quadriceps changes showed the strongest associations, with correlation coefficients ranging from 0.46 to 0.53. Subcutaneous fat prominence demonstrated weaker but still statistically significant correlations. Correlation data are summarized in Table 3.

**Table 3:** Correlation of MRI visual muscle scores with clinical severity measures This table shows Pearson correlation coefficients between MRI-derived visual muscle scores and clinical parameters, including pain intensity and functional limitation, with corresponding p-values indicating statistical significance.

MRI Variable (Visual Score)	Pain VAS (r)		Functional Limitation Score† (r)	p-Value
Quadriceps fatty infiltration	0.46		0.51	<0.001
Quadriceps bulk loss	0.49		0.53	<0.001
Subcutaneous fat prominence	0.22		0.28	0.03

## Discussion

In this study of 150 elderly patients with chronic knee pain, MRI evaluation of both the knee and thigh demonstrated consistent and clinically meaningful patterns of muscle quality changes. The quadriceps muscle group showed the highest degree of fatty infiltration and bulk loss on visual assessment, in agreement with prior work highlighting the vulnerability of the quadriceps in symptomatic knee conditions [1-3]. These findings support the established concept that quadriceps involvement is prominent in chronic knee symptoms, reflecting both disuse and age-related degeneration.

The association between muscle quality and clinical status was clear. Higher visual grades of fatty infiltration and muscle bulk reduction were positively correlated with greater pain severity and poorer functional ability, consistent with earlier MRI-based studies linking intramuscular fat to knee pain and reduced performance [4,5]. Quadriceps changes showed the strongest relationships, emphasizing the central role of this muscle group in knee joint stability, load distribution, and locomotion [6]. Although subcutaneous fat prominence also demonstrated statistically significant correlations, these were comparatively weaker, suggesting that intramuscular changes provide more specific insight into functional decline than superficial fat accumulation.

Using semi-quantitative visual grading rather than specialized quantitative techniques reflects a practical and accessible approach that aligns with routine MRI performed in most clinical settings. Prior literature indicates that visual evaluations can provide clinically meaningful information when applied systematically [7]. These findings confirm that visual assessment can be robust even in centers without access to advanced MRI sequences or post-processing tools [8].

Importantly, the use of combined knee and thigh MRI allowed for comprehensive evaluation of the major muscle groups responsible for joint support and gait mechanics [9-11]. The spectrum of findings across multiple muscle groups suggests that chronic knee pain is associated with broader regional muscle alterations rather than isolated changes localized to the knee.

Overall, this study demonstrates that routine MRI can offer valuable information on muscle quality and may help identify patients at risk for functional decline. The visual grading system used here is feasible, reproducible, and well-suited to daily clinical workflows. Future work should explore whether targeted strengthening or rehabilitation interventions can reverse or stabilize the observed changes and whether muscle quality on MRI can serve as a biomarker for treatment response [12].

Future research directions

Future research in chronic knee pain and fatty muscle changes should focus on establishing a cut-off value for MRI-based muscle degeneration scores to identify patients who would most benefit from physiotherapy interventions. By defining a threshold of fatty infiltration and muscle bulk loss, clinicians could more effectively determine which patients are at higher risk for functional decline and would be ideal candidates for early rehabilitation. Furthermore, longitudinal studies should assess the impact of targeted physiotherapy on muscle quality, specifically investigating whether physiotherapy can reverse or stabilize fatty infiltration and bulk loss in the quadriceps and other key muscle groups. This research could help establish muscle quality as a reliable biomarker for treatment response and optimize rehabilitation strategies for elderly individuals with chronic knee pain.

Strengths and limitations

Strengths of this study include its relatively large sample size, the inclusion of both knee and thigh MRI for comprehensive regional assessment, and the application of a standardized visual grading system. The use of routine MRI sequences enhances the generalizability and clinical applicability of the findings.

Limitations include the cross-sectional design, which does not allow conclusions about causality or the temporal progression of muscle changes. Objective physical activity levels and muscle strength measures were not available, limiting direct biomechanical correlation. Additionally, semi-quantitative visual grading, while practical, may be less sensitive than fully quantitative fat-fraction techniques used in advanced research settings. Finally, the study sample was limited to elderly patients from a single health system, which may affect broader generalizability.

## Conclusions

This study demonstrates that routine knee and thigh MRI provides valuable insight into muscle quality changes among elderly patients with chronic knee pain, with the quadriceps showing the highest levels of fatty infiltration and bulk loss. These semi-quantitative visual findings correlate meaningfully with pain severity and functional limitations, underscoring the central role of regional muscle degeneration in symptom burden. Incorporating systematic assessment of muscle quality into routine knee MRI may help identify patients at higher risk for functional decline and guide early rehabilitative or strengthening interventions.
